# Species composition, abundance and seasonality of potential Rift Valley fever virus vectors in north-eastern KwaZulu-Natal province, South Africa

**DOI:** 10.1186/s13071-026-07564-1

**Published:** 2026-07-18

**Authors:** Takalani I. Makhanthisa, Milehna M. Guarido, Alan Kemp, Melinda K. Rostal, William B. Karesh, Johana Teigen, Assaf Anyamba, Nichar Gregory, Jacqueline Weyer, Peter N. Thompson

**Affiliations:** 1 Department of Production Animal Studies, Faculty of Veterinary Science, University of Pretoria, Onderstepoort, South Africa; 2 Centre for Emerging Zoonotic and Parasitic Diseases, National Institute for Communicable Diseases of the National Health Laboratory Service, Sandringham, South Africa; 3 One Health Research Consulting, Glen Rock, NJ USA; 4 One Health Concepts, Easton, CT USA; 5 University of Maryland Baltimore County, 1000 Hilltop Cir, Baltimore, 21250 USA; 6 Applied Sciences, NASA Goddard Space Flight Centre Earth Science Division, 8800 Greenbelt Rd, Greenbelt, MD 20771 USA; 7 Department of Medical Virology, Faculty of Health Sciences, University of Pretoria, Pretoria, South Africa; 8 Department of Virology, School of Pathology, University of the Witwatersrand, Johannesburg, South Africa; 9 Centre for Veterinary Wildlife Research, Faculty of Veterinary Science, University of Pretoria, Onderstepoort, South Africa

**Keywords:** Mosquito, Rift Valley fever virus, Vector, South Africa

## Abstract

**Background:**

Rift Valley fever virus (RVFV) is endemic in sub-Saharan Africa and the Arabian Peninsula, primarily infecting ruminants such as sheep and cattle and causing high abortion and neonatal mortality rates. Previous studies have indicated hyperendemic circulation of RVFV in north-eastern KwaZulu-Natal (KZN), South Africa. The vectors of RVFV are not well characterized in this area, particularly with respect to species composition, seasonal abundance, and potential vector roles; however, several potential mosquito vector species have been identified. The aim of this study was to describe the species composition, abundance, and seasonal population dynamics of potential RVFV vectors in far north-eastern KZN, in order to identify likely important RVFV vectors in the area.

**Methods:**

Mosquitoes were sampled monthly at three sites (Namaneni, Mpala and Bumbe), for one-to-two nights per site over a period of 3 years (November 2019–November 2022). CO_2_-baited CDC Miniature Light Traps, operated without light, and CO_2_-baited tent traps (modified Shannon) were used at pans (temporary wetlands) at each site. Mosquitoes were morphologically identified using available taxonomic keys. A representative subset (approximately 40%) of collected mosquitoes, including potential primary and secondary vector species, were screened for RVFV RNA using real-time reverse transcription PCR.

**Results:**

A total of 141,594 mosquitoes consisting of 46 species from 8 genera, including *Aedes* (2610), *Culex* (89,143), *Mansonia* (45,029), and others, were collected. *Culex* (*Culex*) *tritaeniorhynchus* (55,491), *Mansonia* (*Mansonioides*) *uniformis* (35,453), and *Culex* (*Lasioconops*) *poicilipes* (14,192) were the most common species, varying in their relative proportions between sites. Mosquito species diversity and abundance differed across sites, with seasonal peaks occurring from January to April. Overall counts were higher at Namaneni (IRR = 3.30, 95% CI 2.62–4.16), *p* < 0.001) but lower at Bumbe (IRR = 0.35, 95% CI 0.23–0.54, *p* < 0.001), compared to Mpala. All 55,113 screened mosquitoes tested negative for RVFV.

**Conclusions:**

Multiple potential RVFV vectors, such as *Cx. tritaeniorhynchus, Cx. poicilipes*, *Aedes* (*Neomelaniconion*) *circumluteolus*, *Aedes* (*Neo.*) *mcintoshi*, and *Aedes* (*Aedimorphus*) *durbanensis*, were found to be present in relatively high numbers and could play an important role in both endemic and epidemic RVFV transmission.

**Graphical Abstract:**

**Figure:**
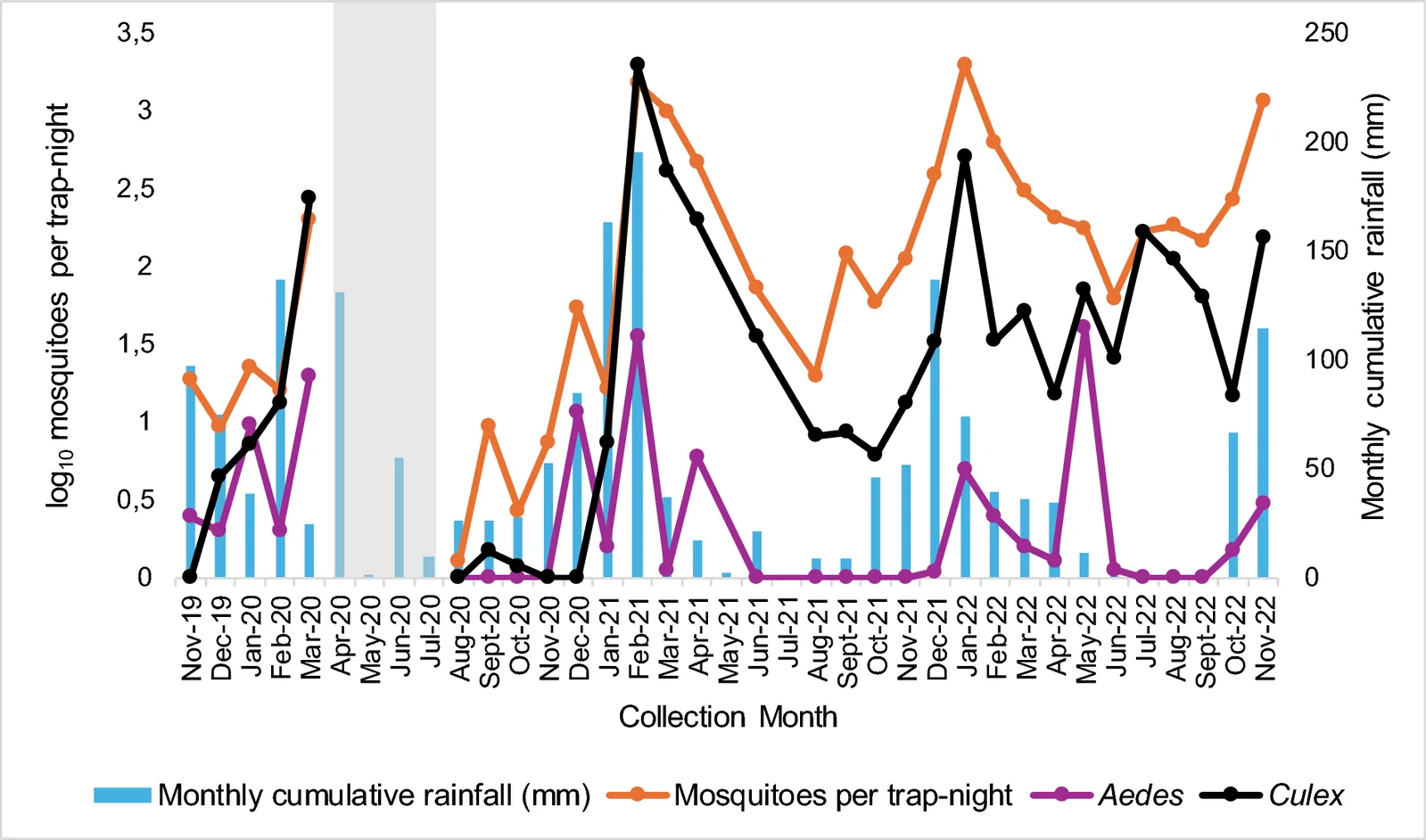

**Supplementary Information:**

The online version contains supplementary material available at https://doi.org/10.1186/s13071-026-07564-1.

## Background

Rift Valley fever (RVF) is a zoonotic, mosquito-borne disease that primarily affects domestic ruminants, such as cattle, goats, and sheep, but can also affect humans mainly via contact with infected animal blood or tissues [1]. Rift Valley fever virus (RVFV) of the genus *Phlebovirus* and family Phenuiviridae [2] is endemic in sub-Saharan Africa and the Arabian Peninsula [3], but has also spread to other African regions [4]. The clinical signs of RVF include high abortion and neonatal mortality rates in ruminants [5]. Most human infections are reported as mild, although a small percentage of patients develop more severe disease, such as encephalitis and hemorrhagic fever [6]. Rift Valley fever virus is believed to be transmitted transovarially through drought-resistant eggs of floodwater-breeding *Aedes* spp. mosquitoes such as those of the subgenera *Neomelaniconion* and *Ochlerotatus* [7, 8], a mechanism thought to contribute to the persistence of RVFV during inter-epidemic periods. These *Aedes* mosquitoes breed in shallow endorheic basins, including pans in South Africa [9] and *dambos* in East Africa [10], which flood after heavy rainfall, leading to the hatching of eggs, some of which may be infected with RVFV [11]. Such floodwater-breeding *Aedes* species are considered important initiators of RVFV transmission following flooding events [11]. Heavy rainfall also favors the breeding of other potential mosquito vectors such as *Culex* spp. [12].

Major outbreaks of RVF in South Africa were reported in 1950–1951, 1974–1976, and 2010–2011 [13]. The first outbreak (1950–1951) was reported in the northern Cape, western Free State and Gauteng (formerly southern Transvaal) provinces [14], leading to a severe outbreak that resulted in an estimated 500,000 abortions and 100,000 deaths in sheep [15]. Larger outbreaks have been reported in the central interior of the country from the Free State, Northern Cape, and Eastern Cape provinces, while smaller outbreaks were noted in KwaZulu-Natal (KZN), Gauteng, and Mpumalanga [13]. The 2010–2011 outbreak resulted in a loss of 19,000 head of livestock and several cases in wildlife [16], and an 8% fatality rate from 302 reported human infections [17]. Outbreaks have not been recorded in Maputaland, a region in the north-east of KZN [13]. However, a recent study reported high RVFV seroprevalence in cattle (34%) and goats (32%) in the area, with a high rate of seroconversion [18], indicating that this is likely a hyperendemic area for RVFV [19]. This suggests ongoing but poorly understood transmission dynamics in the region, highlighting the need for further investigation, and the use of sensitive and reliable tools to detect and monitor circulation. To support such investigations, real-time reverse transcription PCR (RT-PCR) is widely used to detect RVFV in mosquitoes due to its high sensitivity and specificity [20, 21]. 

Rift Valley fever virus has been isolated from various mosquito species which are linked with its maintenance and transmission [12, 22]. *Aedes* (*Neomelaniconion*) *mcintoshi* [23], *Aedes* (*Neomelaniconion*) *circumluteolus*, *Aedes (Aedimorphus) ochraceus*, *Culex* (*Lasioconops*) *poicilipes*, *Culex* (*Culex*) *univittatus*, *Mansonia* (*Mansonioides*) *uniformis*, and *Mansonia* (*Mansonioides*) *africana* are important potential RVFV vectors in East Africa [24]. In West Africa, the virus has been isolated from vectors, such as *Ae. ochraceus*, *Aedes* (*Aedimorphus*) *cumminsii*, *Ae*des (*Aedimorphus*) *vexans*, *Ma. africana*, and *Culex* (*Culex*) *antennatus* [25]. Several species, such as *Cx. antennatus*, *Cx. univittatus*, *Culex* (*Culex*) *pipiens* complex, and *Ma. uniformis*, are potential RVFV vectors in Madagascar [26].

Potential mosquito vectors of arboviruses are species suspected to be capable of transmitting the virus based on their abundance, feeding behavior, presence in areas where the virus occurs or implication as vectors in other areas [27, 28]. Knowledge of RVFV mosquito vectors is important for understanding disease transmission dynamics, prediction of outbreaks, and implementation of effective control strategies [29]. Entomological surveillance and ecological assessments of potential RVFV mosquito vectors are also crucial for evaluating the risk of outbreaks in a specific area and the possibility of the virus spreading to other regions [30, 31]. This highlights the importance of entomological surveillance as a potential early warning tool for RVF outbreaks. To support such surveillance efforts, CO_2_-baited mosquito tent traps and CDC light traps are widely used for sampling mosquito populations and have been shown to be effective in capturing a broad range of species, including potential RVFV vectors [32–34]. These methods are advantageous for field surveillance due to their efficiency in collecting large numbers of mosquitoes and preserving the morphological features necessary for accurate identification [32, 34].

South African transmission studies have shown that *Culex* (*Culex*) *neavei*, *Culex* (*Culex*) *theileri*, *Culex* (*Culex*) *zombaensis*, *Eretmapodites quinquevittatus*, *Ae. circumluteolus*, *Ae. mcintoshi*, and *Cx*. *pipiens* could be infected and successfully transmit RVFV [12, 35]. *Aedes* (*Ochlerotatus*) *caballus* was also reported to be an important RVFV vector in the interior of the country [36]. *Aedes circumluteolus* and *Cx*. *zombaensis* were reported as competent vectors of RVFV in the coastal lowlands of KZN [37]. Other important vectors that have been reported in the region include *Cx. neavei*, *Cx. poicilipes*, and *Cx pipiens* [37, 38]. Recent isolation of RVFV from host-seeking *Aedes* (*Aedimorphus*) *durbanensis* in north-eastern KZN suggests that the species may also be a significant RVFV vector [39]. Based on previous research conducted in that study area, potential RVFV vectors include *Ae. circumluteolus*, *Ae. mcintoshi*, *Ae. durbanensis*, *Ae*. *ochraceus*, *Cx. poicilipes*, and *Culex* (*Culex*) *tritaeniorhynchus* [40].

The aim of this study was to describe the species composition, abundance, and seasonal population dynamics of mosquitoes in Maputaland, north-eastern KZN where evidence suggests endemic RVFV transmission, in order to identify potentially important RVFV vectors. The specific objectives were: 1) to determine the species composition and abundance of potential RVFV vectors in north-eastern KZN, 2) to compare the species composition and abundance of mosquitoes between sites within the study area, 3) to evaluate the influence of climatic variables (rainfall and temperature) on mosquito abundance, and 4) to screen a subset of mosquitoes for RVFV using RT-PCR.

## Methods

### Study area

The Maputaland coastal plain, in far north-eastern KZN, lies south of the Mozambique border and is bounded by the Lebombo Mountains in the west and the Indian Ocean in the east [41]. Maputaland has a humid subtropical climate with dry winters (mean daily temperatures ranging from 16 °C to 26 °C) and hot, rainy summers (mean daily temperatures: 23 °C to 40 °C, mean annual rainfall: 600–800 mm) with the majority of rainfall occurring from October to March [41–43]. The area is characterized by distinct elevated and low-lying topographic features with different soil types, floodplains, and wetland depressions leading to diverse vegetation and high concentration of endemism [44]. The study area features different types of vegetation including floodplain vegetation, bushland, riverine forest, and wooded grassland [45]. The Phongolo river floodplain, at the eastern base of the Lebombo Mountains and covering about 1200 km^2^ [46], is an important part of the study area. Depending on the quantity of rainfall, the Phongolo, Ingwavuma, and Usutu Rivers flood seasonally, covering over 13,000 ha of the floodplain and filling many pans, some of which retain the water during the dry season [47]. The construction of the Pongolapoort Dam in 1973 changed the natural flooding cycle, reducing flood events to instances of heavy rainfall or the occasional opening of the dam’s sluices [48].

**Fig. 1:**
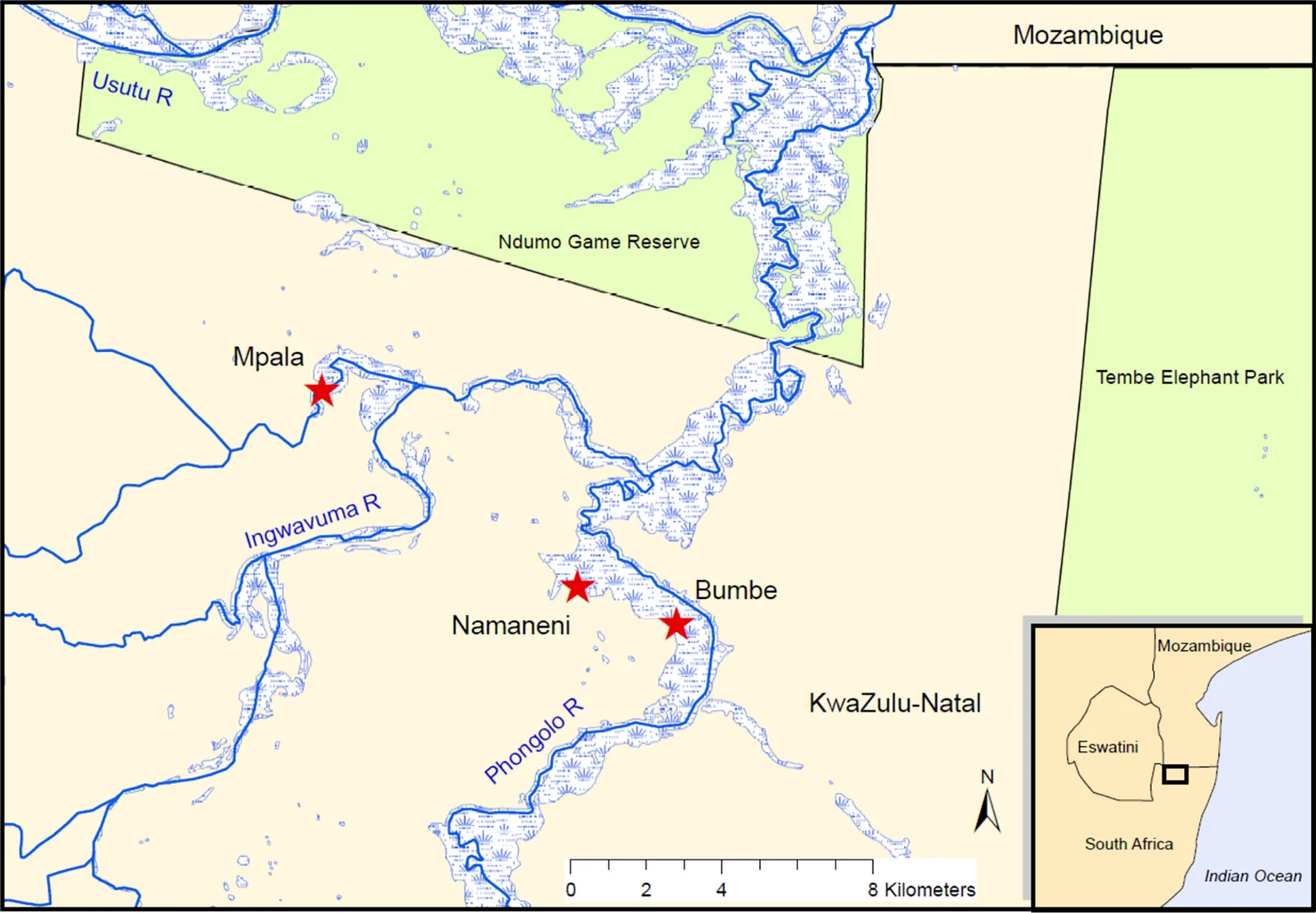
Map of the study area showing the sampling sites in north-eastern KZN. Study sites (Bumbe, Mpala, and Namaneni) are indicated using red stars. The inset map on the bottom right shows the location of the study area within South Africa. The map was generated in ArcGIS version 10.2 (Esri, USA)

The study was conducted at pans in three locations on the floodplain (Fig. 1). The study followed a multi-site longitudinal sampling design with fixed sampling locations. However, sampling periods differed across sites due to logistical and access constraints. Sampling was conducted at Qotho pan on a tributary of the Ingwavuma River, located at Mpala (November 2019–November 2022) and at Phongolo River floodplain pans at Bumbe (November 2019–June 2021) and Namaneni (August 2020–November 2022). The pans are in a communal farming region where cattle and goats graze in large numbers. The study was conducted both during a drought period (2020) when the pans were periodically dry, and after a period of heavy rainfall that occurred in January–February 2021 that led to the pans retaining water for the rest of the sampling period.

### Mosquito collection

Mosquitoes were collected monthly for one-to-two nights over a period of 3 years (November 2019–November 2022). Host seeking female mosquitoes were collected using solid carbon dioxide (CO_2_)-baited mosquito tent (modified Shannon) traps (MTT) and CO_2_-baited CDC Miniature Light Traps (Model 512, BioQuip, USA) operated without the light source, at the two sites (Namaneni and Mpala). To maximize the mosquito catch for virus screening, four CO_2_-baited CDC traps and one CO_2_-baited MTT were used at each site from October 2020 until July 2022. One trap of each type was used per site from November 2019 to September 2020, and again from August 2022 to November 2022. To minimize interference between traps and reduce overlapping attraction ranges, traps were set at least 40 m apart at each site, consistent with spacing used in other mosquito trapping studies in southern Africa [32]. At Bumbe, about 2.5 km from Namaneni and on the same system of interconnected pans, mosquitoes were sampled using one MTT trap, with one CDC trap used from December 2019 to January 2020; however, trapping at this site was stopped in June 2021 due to farming activities that commenced at the site and hindered access. Mosquito traps were set before sunset (15:30–18:30 h) and retrieved in the mornings before sunrise (04:00–6:40 h).

Mosquitoes were removed from MTTs using battery-powered aspirators fitted with labeled collection containers that were then removed and placed in polystyrene boxes containing solid CO_2_ to kill and preserve the mosquitoes. Exposure to solid CO_2_ was kept as brief as possible to minimize potential damage to morphological structures. Specimens were promptly transferred to cold storage to preserve morphological integrity. Mosquitoes trapped in the CDC traps were transferred to the polystyrene boxes with CO_2_ in their respective labeled CDC containers. Dead mosquitoes were transferred to labeled 15 ml or 50 ml Falcon tubes which were transferred to a − 20⁰C portable freezer. Mosquitoes were transported in the freezer to the University of Pretoria, Faculty of Veterinary Science, Department of Veterinary Tropical Diseases, Research and Training Laboratory, where they were stored in a − 20 °C freezer for processing. Mosquitoes were morphologically identified on a chill table under a stereo-microscope to species level using available taxonomic keys [49, 50]. Female mosquitoes were pooled (*n* ≤ 100/pool) according to species, trap type, collection date, site, and blood-fed status, and then stored in labeled tubes at −80ºC for further analysis (virus screening).

### RVF virus screening in mosquitoes

The presence of RVFV was investigated by an RT-PCR method that combined Superscript reverse transcriptase with Taq-polymerase (Roche, USA) and Taqman^®^ probes [20]. Mosquitoes were pooled (*n* ≤ 100) in 2 ml tubes and homogenized in 800 µl Eagle’s Minimum Essential Medium (EMEM) and 2 beads (≥ 25 mosquitoes) or 400 µl EMEM and 1 bead (< 25 mosquitoes). The tubes were bead beaten for 4 min at 30 Hz in a TissueLyser ǁ (QIAGEN, USA) and centrifuged for 2 min at 4722 rpm. RNA was extracted from the samples using AppliedBiosystems MagMax™ Total Nucleic Acid (TNA) extraction kit (Thermo Fisher Scientific, Lithuania) on KingFisher™ Flex instrument (Thermo Fisher Scientific, USA) according to the manufacturer’s instructions.

Real-time RT-PCR was carried out in a LightCycler^®^ 96 (Roche BioSciences) targeting the G2 gene with an amplicon length of 92 bp. Real-time PCR incorporates the use of fluorescent dyes and/or probe to allow real-time detection of the accumulation of PCR products [51]. The forward primer (5′ AAAGGAACAATGGACTCTGGTCA 3′) and reserve primer (5′ CACTTCTTACTACCATGTCCTCCAAT 3′) were used to amplify the gene target as described by Drosten et al. (2002).

### Climate data

Rainfall and temperature data were obtained from a weather station (Davis Weather Instruments, USA) installed at Namaneni in October 2020 for the purpose of this project. Climate data from November 2019 to September 2020 were obtained from a weather station installed at nearby Ndumo Game Reserve to ensure continuous coverage for the full sampling period. Cumulative rainfall (15-day and 30-day) and 15-day average temperature prior to sampling days were calculated from daily records for statistical analyses. For a small number of sampling dates, temperature measurements were unavailable and analyses were conducted using the available data only. The climatic dataset used for the models is provided in Additional file 1_Climate data (Excel).

### Statistical analysis

#### Mosquito abundance

A trap session was defined as a single sampling event conducted on a specific date at a site, during which one or more traps were deployed. Monthly mosquito abundance was standardized as mosquitoes per trap-night to account for differences in trapping effort across sites and sampling periods. This was calculated by dividing the total number of mosquitoes collected per site per month by the total number of traps deployed that month.

#### Species richness and diversity indices

Species richness (S), which is the total number of species observed in an area, and their abundance [52], were calculated for each site. To assess alpha diversity, which is the species diversity within the mosquito communities of the study sites, the Shannon index (H) and Simpson’s index of diversity (1-D) were calculated. The Shannon index accounts for both species richness (number of species) and evenness (relative abundance of each species or homogeneity and heterogeneity of the species), which is the distribution of individuals among species [53–55]. A higher Shannon Index indicates that the community has a more even distribution of species while lower values suggest lower diversity, often indicating dominance by a few species [54, 56]. Simpson's index of diversity reflects the probability that two randomly selected individuals from a community belong to different species [54, 57]. This index produces values ranging from 0 to 1, where a higher value indicates greater species diversity and a more even distribution among species [56]. The Sorenson’s coefficient (CC) was used to assess the beta diversity, which indicates the similarity between the communities at the study sites. It produces values between 0 and 1, where 0 indicates no similarity or shared species and 1 indicates complete similarity or shared species between sites [58].

#### Factors associated with mosquito abundance

Negative binomial regression models were used to assess the associations between mosquito counts and site, trap type, cumulative rainfall (15- or 30-day prior to trapping), average temperature over 15 days prior to trapping, and maximum temperature on the day of capture, with an offset included to account for trap effort (number of trap-nights). Negative binomial models were selected over Poisson regression due to overdispersion in the mosquito count data, where the variance exceeded the mean, violating Poisson model assumptions [59]. The inclusion of trap type as a fixed effect and number of trap-nights as an offset allowed comparisons across sites and sampling periods with unequal trap deployment. This was done for the overall mosquito counts, for each of the major genera (*Aedes*, *Anopheles*, *Culex* and *Mansonia*), and for several species that were well represented or of particular interest as potential RVFV vectors. In each case, the 15-day and 30-day rainfall models were compared using the Akaike information criterion (AIC) [59], and model adequacy was assessed using AIC and inspection of model residuals.

Model results are presented as incidence rate ratios (IRR) with corresponding 95% confidence intervals (CI) and *p *values. The IRR represents the change in the dependent variable (number of mosquitoes caught per trap-night) per unit increase in the predictor variable, with the percentage change calculated as (IRR – 1) × 100 [60].

Statistical analyses were conducted using R (Version 4.2.3) and statistical significance was assessed at *p* < 0.05. The ‘vegan’ package was used for ecological indices, while the ‘MASS’ package was used to fit the negative binomial regression models.

## Results

### Species abundance and composition

A total of 141,594 mosquitoes were trapped over 33 field trips, comprising 139 sampling sessions and 486 trap-nights, across the three study sites (mean per trap-night = 291.3, variance = 402,831). They consisted of 46 species from 8 genera including *Aedes* (2610), *Culex* (89,143), *Mansonia* (45,029), *Anopheles* (4709), *Aedeomyia* (1), *Coquillettidia* (99), *Mimomyia* (2) and *Uranotaenia* (1), and 12 subgenera (Table 1). The most frequently collected species were *Cx*. *tritaeniorhynchus* (*n* = 55,491), *Ma*. *uniformis* (*n* = 35,453), *Cx*. *poicilipes* (*n* = 14,192), and *Cx*. *antennatus* (*n* = 11,838). The most frequently collected *Aedes* species were *Ae. durbanensis* (*n* = 1473), *Ae. mcintoshi* (*n* = 526), and *Ae. circumluteolus* (*n* = 265). The highest number of mosquitoes was caught at Namaneni (53 sessions, *n* = 107,204) compared to Mpala (63 sessions, *n* = 32,213) and Bumbe (23 sessions, *n* = 2177) (Table 1). *Culex tritaeniorhynchus* was the most frequently collected species from all three sites, while floodwater mosquitoes, *Ae. mcintoshi* and *Ae. circumluteolus*, were more common in Mpala and Bumbe than Namaneni (Table 1 and Suppl. Fig. S1). Other potential RVFV vector *Aedes* species that were sampled in the study area included *Ae*des (*Neomelaniconion*) *aurovenatus*, which was only sampled at Namaneni, as well as *Ae*. *ochraceus* and *Ae*. *cumminsi* which were mostly collected at Mpala and Bumbe, respectively (Table 1). Other potential RVFV vector *Culex* species included *Cx*. *neavei*, which was sampled at all three study sites and *Cx*. *zombaensis* which was only present at Mpala and Namaneni (Table 1 and Suppl. Fig. S1).

**Table 1:** Total numbers of mosquitoes collected between November 2019 and November 2022 at three study sites in north-eastern KwaZulu-Natal

**Mosquito species**	**Site** **(number of trap-nights)**			**Total**	**Percentage (%)**
	**Bumbe** **(26)**	**Mpala** **(236)**	**Namaneni** **(224)**		
*Aedeomyia* (*Lep*.) *furfurea*	0	1	0	1	0.001
*Aedes* (*Adm*.) *albocephalus*	0	1	0	1	0.001
*Ae*. (*Adm*.) *argenteopunctatus* group	0	2	0	2	0.001
*Ae*. (*Adm*.) *cumminsi* *	65	1	0	66	0.05
*Ae*. (*Adm*.) *dentatus* group	0	0	1	1	0.001
*Ae*. (*Adm*.) *durbanensis* *	44	282	1,147	1,473	1.04
*Ae*. (*Adm*.)* fowleri*	0	1	10	11	0.01
*Ae*. (*Adm*.)* minutus*	0	1	0	1	0.001
*Ae*. (*Adm*.) *ochraceus* *	0	32	4	36	0.03
*Ae*. (*Adm*.)* unilineatus*	1	0	0	1	0.001
*Ae*. (*Adm*.)* veeniae*	0	1	0	1	0.001
*Ae*. (*Muc*.) *sudanensis*	35	48	42	125	0.09
*Ae*. (*Neo*.) *aurovenatus*	0	0	53	53	0.04
*Ae*. (*Neo*.) *circumluteolus* *	93	100	72	265	0.19
*Ae*. (*Neo*.) *mcintoshi* *	26	483	17	526	0.37
*Ae*. (*Stg*.) *aegypti* *	2	3	0	5	0.004
*Ae*. (*Stg*.) *metallicus*	0	1	0	1	0.001
*Aedes* spp.	1	8	33	42	0.03
*Anopheles* (*Ano*.) *coustani*	96	262	299	657	0.46
*An*. (*Ano*.) *funestus* group	140	24	284	448	0.32
*An*. (*Ano*.) *gambiae* complex	12	45	195	252	0.18
*An*. (*Ano*.)* pharoensis*	11	162	2531	2704	1.91
*An*.(*Ano*.)* pretoriensis*	2	0	0	2	0.001
*An*. (*Ano*.)* rufipes*	24	0	59	83	0.06
*An*. (*Ano*.)* squamosus/cydippis*	24	150	69	243	0.17
*An*. (*Ano*.)* tenebrosus*	0	1	0	1	0.001
*An*. (*Ano*.)* ziemanni*	8	176	52	236	0.17
*Anopheles* spp.	10	15	58	83	0.06
*Coquillettidia* (*Coq*.) *chrysosoma*	0	17	82	99	0.07
*Culex* (*Cux*.) *annulioris*	0	2	1	3	0.002
*Cx.* (*Cux*.) *antennatus* *	0	2844	8994	11,838	8.36
*Cx.* (*Cux*.)* decens/trifoliatus*	0	306	1781	2087	1.47
*Cx*. (*Cux*.)* ethiopicus*	0	0	8	8	0.01
*Cx*. (*Cux*.) *neavei* *	8	100	270	378	0.27
*Cx*. (*Cux*.)* perfuscus*	8	444	2796	3248	2.29
*Cx*. (*Cux*.) *pipiens complex* *	4	307	432	743	0.53
*Cx*. (*Cux*.)* simpsoni*	0	2	3	5	0.004
*Cx*. (*Cux*.)* thalassius*	0	54	28	82	0.06
*Cx*. (*Cux*.)* theileri*	0	5	5	10	0.01
*Cx*. (*Cux*.) *tritaeniorhynchus* *	1,395	18,389	35,707	55,491	39.19
*Cx*. (*Cux*.)* univittatus*	0	19	41	60	0.04
*Cx*. (*Cux*.) *zombaensis* *	0	34	102	136	0.10
*Cx.* (*Las*.) *poicilipes* *	24	4613	9555	14,192	10.02
*Cx*. (*Lut*.) *tigripes*	0	1	1	2	0.001
*Culex* spp.	18	195	647	860	0.61
*Mansonia* (*Man*.) *africana* *	94	590	8225	8909	6.29
*Ma*. (*Man*.) *uniformis* *	32	2430	32,991	35,453	25.04
*Ma*. (*Man*.) spp.	0	60	607	667	0.47
*Mimomyia* (*Mim*.) *mediolineata*	0	0	1	1	0.001
*Mi*. (*Mim*.) *pallida*	0	1	0	1	0.001
*Uranotaenia* spp.	0	0	1	1	0.001
Total	2177	32,213	107,204	141,594	100

^*^ Potential Rift Valley fever virus vector

Abbreviations of subgenera used in this table: *Adm*.   *Aedimorphus*, *Coq*.  *Coquillettidia*, *Las*.   *Lasioconops*, *Lep*.   *Lepiothauma*, *Lut*.   *Lutzia*, *Man*.   *Mansonioides*, *Mim*. *Mimomyia*, *Muc*.   *Mucidus*, *Neo*.   *Neomelaniconion*, *Stg*.   *Stegomyia*

### Species richness and diversity

**Fig. 2:**
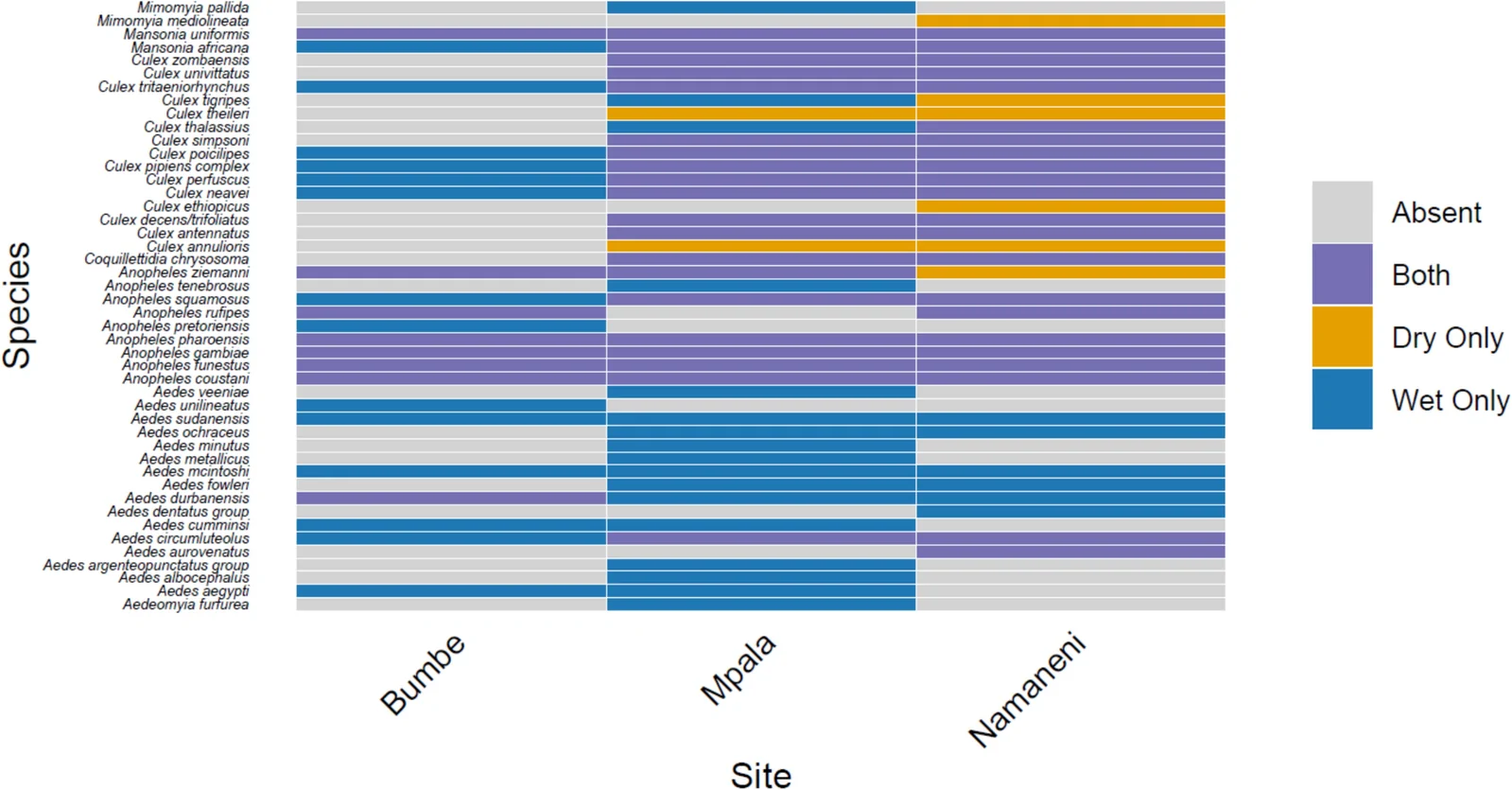
Heatmap of mosquito species occurrence by season (dry = orange, wet = blue, both = purple, absent = gray) across locations (Bumbe, Mpala, and Namaneni). Each row represents a mosquito species and the columns represent sampling locations

Mpala (S = 39) had the highest species richness compared to Namaneni (S = 34) and Bumbe (S = 22). The Shannon and Simpson’s index of diversity showed that Namaneni (H = 1.79 and 1-D = 0.77) had the highest species diversity followed by Mpala (H = 1.56 and 1-D = 0.63), while Bumbe (H = 1.54 and 1-D = 0.57) had the lowest diversity. The Sorensen’s coefficient (CC) showed that community similarities were highest between Namaneni and Mpala (CC = 0.80), while Bumbe shared a 64% and 62% similarity with Mpala and Namaneni, respectively. Total mosquito abundance was higher during the wet season (November–May) than the dry season (June–October) in all study sites (Table 2). Species richness increased in the wet season at Mpala (from 22 to 37 species) and Bumbe (from 8 to 22 species), while remaining constant at Namaneni across seasons (Table 2). Both Shannon (H) and the Simpson’s index of diversity (1-D) showed decreased diversity in the wet season at Mpala and Namaneni but an increase at Bumbe (Table 2). The highest overall diversity (H = 1.97 and 1-D = 0.81) was observed in Namaneni during the dry season (Table 2). Both *Aedes* and *Culex* mosquitoes were more abundant during the wet season across all three sites (Table 2). Namaneni (CC = 0.79) exhibited the highest mosquito species overlap between the two seasons, followed by Mpala (CC = 0.68) and then Bumbe (CC = 0.53). Several potential RVFV vector species including *Cx*. *tritaeniorhynchus*, *Cx*. *antennatus*, and *Ae*. *circumluteolus* were present across seasons at Namaneni and Mpala (Fig. 2). In contrast, potential RVFV vectors, such as *Ae*. *mcintoshi*, *Ae*. *ochraceus*, and *Ae*. *cumminsi*, were detected during the wet season (Fig. 2).

**Table 2:** Species richness (S), diversity indices (Shannon, H and Simpson’s index of diversity, 1-D) and *Aedes* and *Culex* abundance at the three study sites, Bumbe, Mpala, and Namaneni during the wet and dry seasons

**Site**	**Season**	***Total (n)***	***Aedes (n)***	***Culex (n)***	**S**	**H**	**1-D**
Bumbe	Dry	78	1	0	8	1.03	0.47
	Wet	2099	266	1457	22	1.47	0.54
Mpala	Dry	3892	4	2975	22	1.46	0.59
	Wet	28,321	960	24,345	37	1.39	0.55
Namaneni	Dry	10,841	5	4960	28	1.97	0.81
	Wet	96,363	1374	55,411	28	1.68	0.74

Overall, species richness and diversity were higher when assessed using MTT (S = 44, H = 2.08, 1-D = 0.82) compared to CDC (S = 33, H = 1.56, 1-D = 0.70) traps. Species richness and diversity were higher for MTT traps across all sites, with the highest diversity observed at Namaneni (H = 2.03, 1-D = 0.83), while Mpala had the highest species richness (S = 37) (Table 3). The Sorensen’s coefficient (CC = 0.81) between the MTT and CDC showed that their community similarity was 81%.

**Table 3:** Total mosquito abundance, *Aedes* and *Culex* abundance, species richness (S), and diversity indices (Shannon, H and Simpson’s index of diversity, 1-D) for MTT and CDC traps across the three sampling sites

**Site**	**Trap**	***Total (n)***	***Aedes (n)***	***Culex (n)***	**S**	**H**	**1—D**
Bumbe	CDC	70	5	2	5	0.76	0.37
	MTT	2107	262	1455	22	1.47	0.54
Mpala	CDC	20,679	234	17,752	28	1.41	0.62
	MTT	11,534	730	9590	37	1.71	0.65
Namaneni	CDC	66,546	826	38,004	29	1.54	0.70
	MTT	40,658	553	22,367	34	2.03	0.83

### Effects of site, trap type, and climatic variables on mosquito abundance

**Fig. 3:**
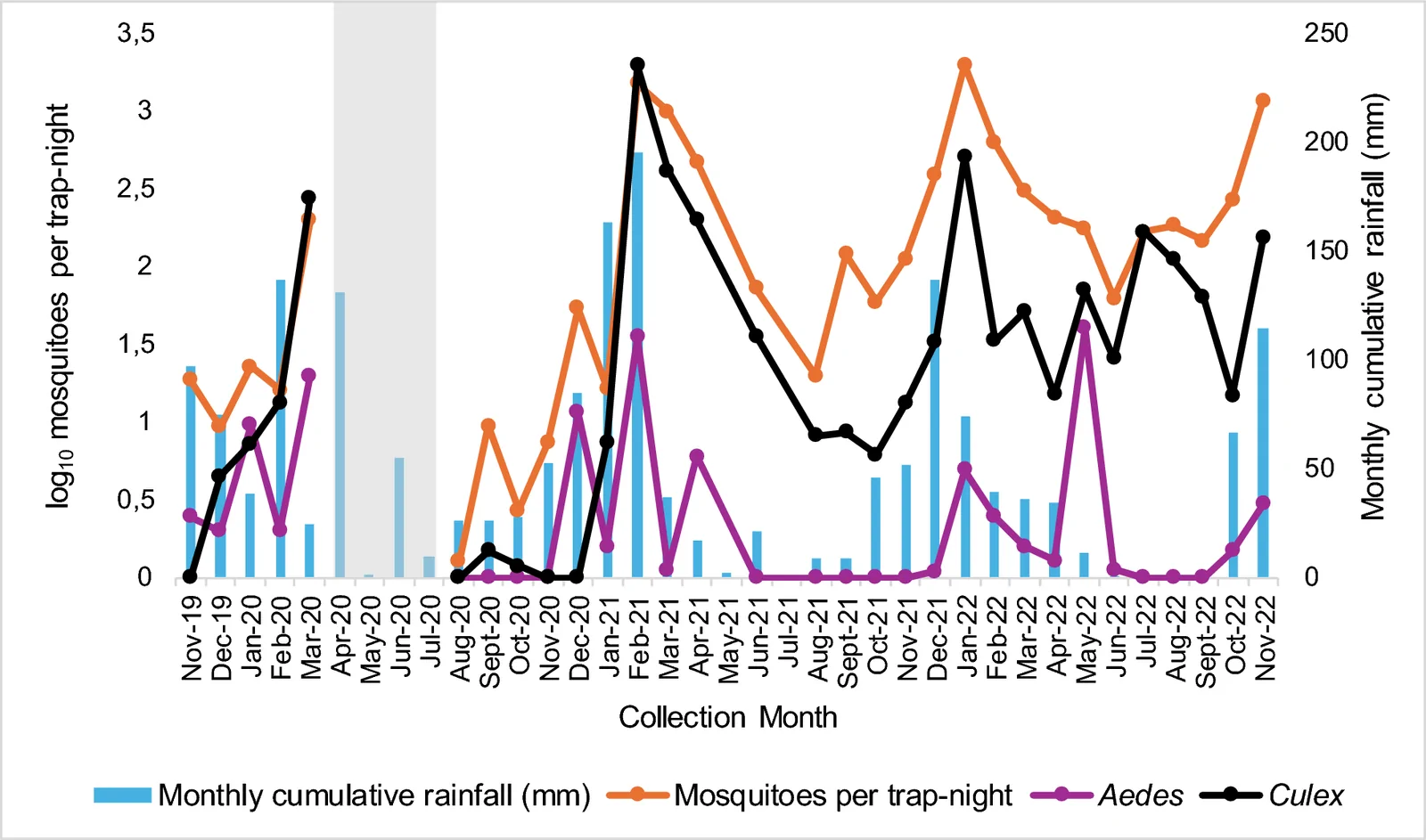
Number of mosquitoes (orange), *Aedes* (purple), *Culex* (black) collected per trap-night plotted on a log_10_(count + 1) scale, and total monthly rainfall (blue bars) over the 3-year collection period in north-eastern KwaZulu-Natal. The shaded gray box indicates the period of no sampling (April–July 2020) due to COVID-19 restrictions. Raw mosquito counts per trap night and monthly cumulative rainfall data are provided in Suppl. Table S1

The total rainfall recorded during the 3-year sampling period (November 2019–November 2022) was 1863 mm, with a mean (± SD) monthly rainfall of 50.3(± 50.8) mm. The mosquito counts increased markedly after the heavy rainfalls that occurred in January (163.4 mm) and February 2021 (195.4 mm) (Fig. 3). The total number of mosquitoes collected per trap-night increased from January 2021 (*n* = 15.6) to February 2021 (*n* = 1526.6) (Fig. 3 and Suppl. Table S1). The heavy rainfall in January and February 2021 led to a rapid increase in *Culex* spp. across all sites, while the abundance of *Aedes* spp. and *Anopheles* spp. remained relatively low (Suppl. Fig. S2). The *Aedes* peak (*n* = 475) observed in May 2022 (Fig. 3), comprised only of *Ae*. (*Neomelaniconion*) spp.. Compared to Mpala and Bumbe, *Mansonia* spp. were more abundant at Namaneni, with a peak observed in January–February 2022 (Suppl. Fig. S2).

In the negative binomial regression models, model selection was based on AIC values, comparing models fitted with either 15-day or 30-day cumulative rainfall while holding other predictors constant. AIC values for all mosquito groups are presented in suppl. Table S2. Models with 30-day cumulative rainfall was of better fit (lower AIC) for predicting the overall number of mosquitoes caught, as well as the *Aedes* spp., *Anopheles* spp., *Culex* spp., and *Ae*. (*Neomelaniconion*) spp. counts (suppl. Table S2). The models incorporating 15-day cumulative rainfall provided a better fit for predicting the abundance of *Mansonia* spp., *Ae*. *durbanensi*s, *Cx*. *antennatus*, *Cx*. *neavei*, *Cx*. *poicilipes*, and *Cx*. *tritaeniorhynchus* (suppl. Table S2).

The overall mosquito abundance was higher at Namaneni (IRR = 3.30, 95% CI 2.62–4.16, *p* < 0.001) but lower at Bumbe (IRR = 0.35, 95% CI 0.23–0.54, *p* < 0.001) compared to Mpala (Table 4). Relative to Mpala, *Aedes* spp. abundance did not differ significantly at either Bumbe (IRR = 0.58, 95% CI: 0.20–1.71, *p* = 0.324) or Namaneni (IRR = 0.68, 95% CI 0.37–1.27, *p* = 0.225). Compared to Mpala, abundances of *Anopheles* spp. (IRR = 4.58, 95% CI 3.25–6.44, *p* < 0.001), *Culex* spp. (IRR = 2.16, 95% CI 1.50–3.12, *p* < 0.001) and *Mansonia* spp. (IRR = 9.18, 95% CI 6.01–14.02, *p* < 0.001) were higher at Namaneni. At Bumbe, *Anopheles* spp. were also more abundant (IRR = 1.95, 95% CI 1.06–3.59, *p* = 0.032), whereas *Culex* spp. and *Mansonia* spp. decreased (IRRs < 1, *p* < 0.05) (Table 4). *Culex antennatus* abundance was markedly higher at Namaneni (IRR = 6.29, 95% CI 3.10–12.78, *p* < 0.001) compared to Mpala, while it was not present in samples from Bumbe. The abundance of *Cx*. *poicilipes* (IRR = 2.44, 95% CI 1.42–4.19, *p* < 0.001) was also higher at Namaneni compared to Mpala. No significant differences in abundance were observed at other sites for *Ae*. (*Neomelaniconion*) spp., *Ae*. *durbanensis*, *Cx*. *neavei*, and *Cx*. *tritaeniorhynchus* compared to Mpala (Table 5).

**Table 4:** Negative binomial regression results showing the associations of site, trap type, rainfall and temperature with the number of mosquitoes caught at three sites in north-eastern KwaZulu-Natal

**Variable**	**Overall count**		***Aedes***		***Anopheles***		***Culex***		***Mansonia***	
	**IRR (95% CI)**	***p***	**IRR (95% CI)**	***p***	**IRR (95% CI)**	***p***	**IRR (95% CI)**	***p***	**IRR (95% CI)**	***p***
Site										
Mpala	1*	-	1*	-	1*	-	1*	-	1*	-
Bumbe	0.35(0.23–0.54)	< 0.001	0.58(0.20–1.71)	0.324	1.95(1.06–3.59)	0.032	0.29(0.15–0.56)	< 0.001	0.23(0.10–0.52)	< 0.001
Namaneni	3.30(2.62–4.16)	< 0.001	0.68(0.37–1.27)	0.225	4.58(3.25–6.44)	< 0.001	2.16(1.50–3.12)	< 0.001	9.18(6.01–14.02)	< 0.001
Trap type										
CDC	1*	-	1*	-	1*	-	1*	-	1*	-
MTT	1.65(1.31–2.07)	< 0.001	6.17(3.35–11.38)	< 0.001	6.25(4.46–8.75)	< 0.001	1.77(1.23–2.53)	0.002	0.72(0.47–1.09)	0.119
15-d rainfall										
< 10 mm									1*	-
10–50 mm									0.83(0.53–1.31)	0.428
> 50 mm									5.59(2.64–11.83)	< 0.001
30-d rainfall										
< 30 mm	1*	-	1*	-	1*	-	1*	-		
30–100 mm	0.82(0.62–1.09)	0.178	28.24(12.89–61.88)	< 0.001	0.37(0.24–0.57)	< 0.001	0.54(0.34–0.85)	0.007		
> 100 mm	4.15(2.82–6.12)	< 0.001	51.92(18.0–149.76)	< 0.001	0.75(0.42–1.33)	0.322	3.58(1.93–6.12)	< 0.001		
15-d temperature	1.11(1.04–1.18)	0.001	0.70(0.59–0.84)	< 0.001	0.91(0.83–1.00)	0.046	1.23(1.11–1.36)	< 0.001	1.21(1.09–1.33)	< 0.001
Maximum temperature on trap day	1.00(0.98–1.03)	0.777	1.35(1.24–1.46)	< 0.001	1.03(0.99–1.08	0.177	0.94(0.90–0.99)	0.011	1.00(0.95–1.05)	0.988

1* = Reference category, IRR = Incidence rate ratios with 95% confidence intervals (CI) and *p* values, 15-d rainfall = 15-day cumulative rainfall (categories: < 10 mm, 10–50 mm and > 50 mm) prior to day of capture, 30-d rainfall = 30-day cumulative rainfall (categories: < 30 mm, 30–100 mm and > 100 mm) prior to day of capture, 15-d temperature = 15-day average temperature prior to day of capture

**Table 5:** Negative binomial regression results showing the associations of site, trap type, rainfall, and temperature with the number of mosquitoes of some potential Rift Valley fever virus vector species caught at three sites in north-eastern KwaZulu-Natal

	***Ae. (Neomelaniconion) spp.***		***Ae. durbanensis***		***Cx. antennatus***		***Cx. neavei***		***Cx. poicilipes***		***Cx. tritaeniorhynchus***	
	**IRR (95% CI)**	***p***	**IRR (95% CI)**	***p***	I**RR (95% CI)**	***p***	**IRR (95% CI)**	***p***	**IRR(95%CI)**	***p***	**IRR (95% CI)**	***p***
Site												
Mpala	1*	-	1*	-	1*	-	1*	-	1*	-	1*	-
Bumbe	0.47(0.11–1.98)	0.300	0.29(0.02–4.01)	0.357	0.00(0.00-inf)	0.000	0.25(0.03–1.94)	0.186	0.07(0.02–0.19)	< 0.001	0.42(0.09–2.07)	0.287
Namaneni	0.43(0.18–1.02)	0.055	0.68(0.16–2.94)	0.607	6.29(3.10–12.78)	< 0.001	2.47(0.92–6.61)	0.072	2.44(1.42–4.19)	0.001	1.30(0.52–3.26)	0.569
Trap type												
CDC	1*	-	1*	-	1*	-	1*	-	1*	-	1*	-
MTT	7.08(3.03–16.52)	< 0.001	3.77(0.88–16.22)	0.074	2.07(1.03–4.20)	0.042	12.68(4.76–33.76)	< 0.001	1.60(0.94–2.72)	0.084	1.32(0.54–3.24)	0.545
15-d rainfall												
< 10 mm			1*	-	1*	-	1*	-	1*	-	1*	-
10–50 mm			22.08(4.43–109.95)	< 0.001	0.19(0.08–0.42)	< 0.001	0.15(0.05–0.46)	< 0.001	0.58(0.33–1.04)	0.068	35.86(13.82–93.06)	< 0.001
> 50 mm			4.43(0.48–40.98)	0.190	33.39(9.46–117.89)	< 0.001	1.33(0.24–7.30)	0.746	3.23(1.24–8.42)	0.016	5.45(1.12–26.46)	0.035
30-d rainfall												
< 30 mm	1*	-										zz
30–100 mm	67.17(17.80–253.43)	< 0.001										
> 100 mm	433.94(77.57–2427.48)	< 0.001										
15-d temperature	0.65(0.51–0.84)	< 0.001	1.01(0.69–1.48)	0.963	1.12(0.93–1.32)	0.265	1.28(1.01–1.62)	0.041	1.02(0.90–1.16)	0.738	1.52(1.23–1.88_	< 0.001
Maximum temperature on trap day	1.03(0.93–1.15)	0.567	2.02(1.63–2.50)	< 0.001	0.75(0.68–0.83)	< 0.001	0.77(0.68–0.89)	< 0.001	0.93(0.86–0.99)	0.024	1.30(1.16–1.46)	< 0.001

1* = Reference category, IRR = Incidence rate ratios with 95% confidence intervals (CI) and *p* values, 15-d rainfall = 15-day cumulative rainfall (categories: < 10 mm, 10–50 mm and > 50 mm) prior to day of capture, 30-d rainfall = 30-day cumulative rainfall (categories: < 30 mm, 30–100 mm and > 100 mm) prior to day of capture, 15-d temperature = 15-day average temperature prior to day of capture

Overall mosquito counts (IRR = 1.65, 95% CI 1.31–2.07), *p* < 0.001), *Aedes* spp. (IRR = 6.17, 95% CI 3.35–11.38, *p* < 0.001), *Anopheles* spp. (IRR = 6.25, 95% CI 4.46–8.75, *p* < 0.001), and *Culex* spp. (IRR = 1.77, 95% CI: 1.23–2.53, *p* = 0.002) counts were higher in MTT traps compared to CDC traps (Table 4). However, trap type was not significantly associated with counts of *Mansonia* spp. (IRR = 0.72, 95% CI 0.47–1.09, *p* = 0.119) (Table 4). MTT traps captured markedly higher numbers of *Ae*. (*Neomelaniconion*) spp. (IRR = 7.08, 95% CI 3.03–16.52, *p* < 0.001) and *Cx*. *neavei* (IRR = 12.68, 95% CI 4.76–33.76, *p* < 0.001) compared to CDC traps (Table 5). No significant associations with trap type were observed for *Ae*. *durbanensis*, *Cx*. *poicilipes*, or *Cx*. *tritaeniorhynchus* (Table 5).

Increased 30-day cumulative rainfall had a very large positive effect on *Aedes* spp. counts for both 30–100 mm (IRR = 28.24, 95% CI 12.89–61.88, *p* < 0.001) and > 100 mm (IRR = 51.92, 95% CI 18.0–149.76, *p* < 0.001) rainfall categories. However, while 30-day cumulative rainfall > 100 mm was also associated with increased overall mosquito (IRR = 4.15, 95% CI 2.82–6.12, *p* < 0.001) and *Culex* spp. abundance (IRR = 3.58, 95% CI 1.93–6.12, *p* < 0.001), rainfall between 30 and 100 mm had a somewhat negative effect on *Culex* spp. (IRR = 0.54, 95% CI 0.34–0.85, *p* = 0.007) and *Anopheles* spp. (IRR = 0.37, 95% CI 0.24–0.57, *p* < 0.001) (Table 4). Higher 15-day cumulative rainfall (> 50 mm) was positively associated with *Mansonia* spp. counts (IRR = 5.59, 95% CI 2.64–11.83, *p* < 0.001), while lower rainfall amounts (< 50 mm) showed no significant effect (Table 4). Higher 30-day cumulative rainfall was associated with a large increase in the abundance of *Ae*. (*Neomelaniconion*) spp. for both 30–100 mm (IRR = 67.17, 95% CI 17.80–253.43, *p* < 0.001) and > 100 mm (IRR = 433.94, 95% CI 77.57–2427.48, *p* < 0.001) rainfall categories (Table 5). For 15-day cumulative rainfall, large increases were observed for *Ae*. *durbanensis* (IRR = 22.08, 95% CI 4.43–109.95, *p* < 0.001) and *Cx*. *tritaeniorhynchus* (IRR = 35.89, 95% CI 13.82–93.06, *p* < 0.001) for the 10–50 mm category. A large positive effect was observed for the 15-day cumulative rainfall > 50 mm category for *Cx*. *antennatus* (IRR = 33.39, 95% CI 9.46–117.89, *p* < 0.001) and *Cx*. *tritaeniorhynchus* (IRR = 5.45, 95% CI 1.12–26.46, *p* = 0.035), while *Cx*. *poicilipes* (IRR = 3.23, 95% CI 1.24–8.42, *p* = 0.016) showed a moderate positive association (Table 5).

Higher 15-day average temperature prior to the day of capture was associated with increased overall (IRR = 1.11, 95% CI 1.04–1.18, *p* = 0.001), *Culex* spp. (IRR = 1.23, 95% CI 1.11–1.36, *p* < 0.001), *Mansonia* spp. (IRR = 1.21, 95% CI:1.09–1.33, *p* < 0.001), *Cx*. *neavei* (IRR = 1.28, 95% CI 1.01–1.62, *p* = 0.041), and *Cx*. *tritaeniorhynchus* (IRR = 1.52, 95% CI 1.23–1.88, *p* < 0.001) counts (Tables 4 and 5). For *Aedes* spp. (IRR = 0.70, 95% CI 0.59–0.84, *p* < 0.001), *Anopheles* spp. (IRR = 0.91 95% CI 0.83–1.00, *p* = 0.046) and *Ae*. (*Neomelaniconion*) spp. (IRR = 0.65, 95% CI 0.51–0.84, *p* < 0.001, higher 15-day average temperature was associated with lower counts (Tables 4 and 5). No significant associations were observed between 15-day average temperature and counts of *Ae*. *durbanensis*, *Cx*. *antennatus*, and *Cx*. *poicilipes* (Table 5).

Maximum daily temperature on the day of capture was positively associated with counts of *Aedes* spp. (IRR = 1.35, 95% CI 1.24–1.46, *p* < 0.001), *Ae*. *durbanensis* (IRR = 2.02, 95% CI 1.63–2.50, *p* < 0.001), and *Cx*. *tritaeniorhynchus* (IRR = 1.30, 95% CI 1.16–1.46, *p* < 0.001) (Tables 4 and 5). In contrast, *Culex* spp. (IRR = 0.94, 95% CI 0.90–0.99, *p* = 0.011), *Cx. antennatus* (IRR = 0.75, 95% CI 0.68–0.83, *p* < 0.001), *Cx*. *neavei* (IRR = 0.77, 95% CI 0.68–0.89, *p* < 0.001), and *Cx*. *poicilipes* (IRR = 0.93, 95% CI 0.86–0.99, *p* = 0.024) were negatively influenced by increased maximum temperature on the day of capture (Tables 4 and 5). No significant associations were observed between the maximum daily temperature and the overall mosquito abundance, or the abundance of *Anopheles* spp., *Mansonia* spp., or *Ae*. (*Neomelaniconion*) spp. (Tables 4 and 5).

### RVFV screening in mosquitoes

A total of 55,113 mosquitoes grouped in 1074 pools were screened for RVFV using RT-PCR. All pools tested negative for RVFV. Mosquito species data for all screened specimens are provided in Suppl. Table S3.

## Discussion

This study assessed mosquito communities at three sites in north-eastern KZN, where recent endemic circulation of RVFV with high seroprevalence was detected [18], to assess the species composition, seasonality, and predictors of abundance of potential RVFV vectors. Rift Valley fever outbreaks have not been documented in the area, which could be attributed to the herd immunity arising from frequent exposure to the virus, thereby lowering the likelihood of identifiable outbreaks [19, 61]. Given the presence of known and potentially competent mosquito vectors, this region could act as a source of RVFV introduction into non-endemic areas, where livestock populations may be more susceptible. Identifying vector communities provides important ecological insights into RVF epidemiology and the risk of RVFV spread to other areas.

Overall mosquito counts were highest between January and March, corresponding to peak summer rainfall and temperature conditions. Similar seasonal mosquito peaks have been reported for other RVFV-endemic regions, where increased rainfall and favorable climatic conditions promote breeding site availability [62]. Several potential vector species, including *Cx*. *tritaeniorhynchus*, *Cx*. *antennatus*, and *Ae*. *circumluteolus*, were present at Namaneni and Mpala across all seasons, indicating that competent vectors persist outside the main rainy season in this hyperendemic region, and could potentially facilitate virus circulation throughout the year [19]. In contrast, potential RVFV vectors, such as *Ae*. *mcintoshi*, *Ae*. *ochraceus*, and *Ae*. *cumminsi*, were only detected during the wet season. This is consistent with studies that have shown that abundances of such floodwater mosquito species elsewhere increase with rainfall and flooding, especially in epidemic-prone areas [24, 63].

The most abundant mosquito species we collected were *Cx. tritaeniorhynchus*, *Ma. uniformis*, and *Cx. poicilipes*, which were all collected in relatively higher numbers at Namaneni compared to Mpala and Bumbe. These vectors were also found to be the most abundant species in an RVFV ecological study (2014–2015) conducted in Senegal [62]. *Culex tritaeniorhynchus* typically breed in permanent or semi-permanent freshwater water bodies colonized by aquatic plants [62]. Similarly, *Mansonia* spp. breed in freshwater containing floating vegetation [64]. These habitats occur within the study area and may have contributed to the abundance of these species.

*Culex tritaeniorhynchus* was confirmed as a vector of RVFV during the 2000 RVF epidemic in Saudi Arabia, based on virus isolations, vector competence tests, abundance, and preference for feeding on humans and sheep [65]. Historically, *Cx*. *tritaeniorhynchus* was not collected in large numbers in the southern African subregion, and prior to the Saudi Arabian RFV outbreak, had not been implicated as a vector of RVFV [65]. However, this study shows that this species currently occurs at high abundances in north-eastern KZN, and may represent an important potential RVFV vector in the area. *Culex poicilipes* has previously been shown experimentally to transmit RVFV efficiently at rates of 15% by day 15 and 80% by day 30 post-infection of the mosquito [38]. Additionally, RVFV was isolated from 63 and 36 pools of naturally infected *Cx. poicilipes* from Senegal and Mauritania, respectively, during the 1998–1999 RVFV outbreak [66]. In our study, *Cx. tritaeniorhynchus* increased under moderate (10–50 mm) and high (> 50 mm) 15-day rainfall, while *Cx*. *poicilipes* declined at moderate rainfall but increased at higher levels. These patterns suggest that their roles as RVFV vectors may shift under different rainfall conditions.

*Mansonia uniformis* and *Ma. africana* have also been implicated in RVFV transmission, with RVFV RNA detected by PCR in *Ma. africana* collected during an outbreak in Kenya [24, 67] and in *Ma. uniformis* sampled during the 2006–2007 East African outbreak [21]. Their abundance in the study area suggests that they may also contribute to RVFV transmission. However, vector competency tests are required to confirm these mosquito species as efficient vectors [27].

In contrast to the most abundant species sampled during the study, the floodwater *Ae*. (*Neomelaniconion*) spp., *Ae. mcintoshi*, and *Ae. circumluteolus*, were mostly collected at Mpala. These species increased significantly with higher 30-day cumulative rainfall, with substantial rises observed at both 30–100 mm and > 100 mm rainfall categories. This strong positive response to rainfall is consistent with the known association of these species with temporary floodwater habitats. Floodwater *Aedes* spp., mainly from the subgenera *Aedimorphus* and *Neomelaniconion*, lay drought-resistant eggs on the edges of low-lying grasslands or shallow topographical depressions (dambos/pans) that are prone to flooding [23]. The eggs can withstand desiccation and become dormant until flooding, which triggers them to hatch [23]. Eggs may be infected with RVFV, via transovarial transmission which may contribute to the maintenance of RVFV persistence in the environment [8]. The significant rise in abundance of *Ae*. (*Neomelaniconion*) spp. observed in May 2022 in our study area suggests that they may be able to transmit RVFV well beyond the main rainy season. These species have been reported to be widely distributed in South Africa, particularly in the Lowveld (hot, low-lying northern and eastern regions), Highveld (cooler, high-altitude plateau), Karoo (semi-arid central interior), and the coast of KZN [68–70]. *Aedes mcintoshi* has been recognized as the primary vector in inland regions, and *Ae. circumluteolus* in coastal KZN [7]. In our study, however, *Ae. mcintoshi* was collected more frequently than *Ae. circumluteolus*, suggesting a possible change in species composition in the region and indicating that *Ae. mcintoshi* may also contribute to local RVFV transmission, including transovarially.

The other relatively abundant aedine species, *Ae. durbanensis*, was positively influenced by 15-day rainfall (10–50 mm) and increased daily maximum temperature. However, the positive influence by higher maximum temperature on the day of capture, likely reflect increased adult mosquito activity and host-seeking behavior, rather than a true increase in population size [71]. Although RVFV vector competency tests have not been conducted for *Ae. durbanensis*, the virus was recently detected in a pool of *Ae. durbanensis* (4,077 mosquitoes screened across 105 pools) in the study area [39] and its abundance at times during the current study further suggests that it could serve as a potential RVFV vector. Rainfall supports mosquito breeding by providing aquatic habitats; however, excessive or prolonged rainfall can disrupt these sites, washing away or directly killing immature stages [72]. Most *Ae. durbanensis* (79.3%) were collected prior to the flooding that occurred in January and February 2021, suggesting that heavy rainfall may have reduced their populations. This pattern may also reflect an initial mass hatching when wetlands first filled, with remaining viable eggs of this floodwater species potentially requiring a drying period before hatching after subsequent floods [23].

*Culex antennatus*, which is recognized as an RVFV vector [73, 74], was sampled in relatively high numbers (8%) and responded positively to higher 15-day cumulative rainfall (> 50 mm), suggesting that this climatic condition may favor environmental conditions associated with increased abundance. Mosquito population dynamics are shaped by multiple interacting factors, including the availability of suitable aquatic habitats and vegetation [75]. *Culex antennatus* has demonstrated RVFV vectorial competence [22, 74, 76] and has been implicated in outbreaks in Egypt [73], Madagascar [77], and Mauritania [78]. Its high abundance in this study suggests that it could play a role as an RVFV vector in the study area.

*Culex zombaensis*, which was proven to be a competent vector during an RVF outbreak in the coastal KZN in 1981 [37], was collected in lower numbers (0.1%) in the current study. *Culex zombaensis* was also implicated in an outbreak of RVFV among domestic animals in Kenya [67], while *Cx. neavei* was identified as a vector in Mauritania [78]. *Culex neavei*, from which RVFV was also isolated during the 1981 outbreak in coastal KZN [37], constituted 0.27% of the collections in our study. Other potential RVFV vectors, including *Cx. pipiens* complex, *Ae. cumminsi*, *Ae. aegypti*, and *Ae. ochraceus,* which have been implicated in RVFV transmission in other areas [8, 24, 29, 79] were also collected in the current study, although in lower numbers.

Although Mpala had the highest species richness (S = 39), both the Shannon Index (H) and the Simpson’s index of diversity (1-D) showed that species diversity was highest at Namaneni (H = 1.79 and 1-D = 0.77). The ecological indices from all study sites showed moderate diversity (H between 1.5 and 3.5) [54, 80], with a tendency toward dominance by a few species. The unbalanced mosquito populations were mostly caused by *Cx. tritaeniorhynchus*, which was the dominant species for all study sites. The level of mosquito diversity indicated by the two biodiversity indices in this study is comparable to those in mosquito ecological studies conducted in other parts of South Africa such as Tshokwane (H = 1.71), Skukuza (H = 1.80), and Lower Sabie (H = 1.76) in the Kruger National Park, as well as at Lapalala Nature Reserve (H = 1. 99 and 1-D = 0.74) [81, 82]. The mosquito communities of Namaneni and Mpala had a similarity rate of 80%, which was expected because of similarities between the habitats and the overlapping sampling periods. One limitation of the study was that mosquito trapping at Bumbe occurred over a shorter duration (November 2019–June 2021) with fewer traps per night due to farming activities, which limited both the abundance and diversity of collected mosquitoes. In addition, differences in the proportional use of trapping methods between sites, particularly the use of predominantly MTT traps at Bumbe, may have influenced the composition of mosquito species collected and therefore represent a limitation when comparing mosquito communities among sites. The pans at both Namaneni and Mpala retained water after the 2021 (January–February) rainfall, which could have influenced similarities in community composition, particularly through increases in *Culex* spp. abundance, by providing extended breeding opportunities for mosquitoes in long-standing water. Mosquito populations are also influenced by availability of blood meal sources. Potential vectors of RVFV have been reported to feed on hosts, such as humans, cattle, goats, sheep, and donkeys [83, 84]. Diverse wild and domestic mammal and bird species were available in the study area [84], and this could also have influenced the observed mosquito population diversity.

The two different mosquito trapping methods used in this study influenced the abundance and diversity of mosquito populations collected. Both CO_2_-baited MTT and CDC traps have been proven to be among the most efficient methods for sampling mosquito populations [32]. Overall, the MTT traps collected higher mosquito numbers and greater species diversity than CDC traps. Another key benefit of MTTs is that mosquitoes can be collected with minimal damage, facilitating morphological identification [85] compared to the CDC that contains motorized parts. However, a major advantage of CDC traps is their flexibility in retrieval timing, whereas MTT traps must be cleared by sunrise.

No RVFV was detected from the 55,113 mosquitoes that were screened in 1,074 pools. Low detection rates of RVFV in mosquitoes have been reported for species such as *Cx. antennatus* (isolation from 2 pools out of 319 pools) [77], *Cx. zombaensis* (5 isolations from 18,831 mosquitoes), and *Ma. africana* (1 isolation from 14,439 mosquitoes) [67]. Other studies conducted previously have failed to detect RVFV in mosquitoes [31, 33], making the results of this study comparable. With endemic rather than epidemic RVFV transmission expected in this area, the infection rate in mosquitoes is likely to be very low.

The data generated in this study can be used to enhance the accuracy of RVF outbreak prediction systems that are guided by monitoring climate and vegetation [86, 87]. Current RVFV early warning systems do not account for the timing and abundance of primary vector species, which are critical factors for virus emergence and amplification [87]. Identifying periods of high abundance of RVFV vector species is important for understanding periods of elevated transmission risk [30]. In addition, vector community analysis, considering factors, such as relative abundances, seasonal composition, and ecological traits [88], provides ecological insights into RVFV, improves our understanding of RVFV reservoir systems [19] and may help improve climate-based prediction models.

## Conclusions

This study showed that several mosquito species that have been implicated in RVFV transmission, including *Cx. tritaeniorhynchus*, *Cx. poicilipes*, *Cx. antennatus*, and *Mansonia* spp., occur in high abundance in north-eastern KZN and could play an important role in endemic RVFV transmission. In addition, the presence of floodwater *Aedes* mosquitoes, including *Ae. circumluteolus* and *Ae. mcintoshi*, which are believed to be maintenance vectors of RVFV through transovarial and cryptic horizontal transmission, were also present. RVFV vector species were detected throughout the year. Given that RVFV transmission involves multiple vector species, the use of MTT traps could improve surveillance and provide a more comprehensive assessment of mosquito communities in the area. The influence of climatic factors such as rainfall and temperature on potential RVFV mosquito vector population dynamics may contribute to systems used to predict future RVF outbreaks. This research will contribute to the limited current knowledge on RVFV vector population dynamics and mosquito ecology in north-eastern KZN and likely also in similar RVFV-endemic regions.

## Supplementary Information

Supplementary Material 1

## Data Availability

All data generated or analyzed during this study are included in this article and its additional files.
